# Influence of Geometrical Shape on the Characteristics of the Multiple InN/In*_x_*Ga_1−*x*_N Quantum Dot Solar Cells

**DOI:** 10.3390/nano11051317

**Published:** 2021-05-17

**Authors:** Asmae El Aouami, Laura M. Pérez, Kawtar Feddi, Mohamed El-Yadri, Francis Dujardin, Manuel J. Suazo, David Laroze, Maykel Courel, El Mustapha Feddi

**Affiliations:** 1 Group of Optoelectronic of Semiconductors and Nanomaterials, ENSAM, Mohammed V University in Rabat, Rabat 10100, Morocco; Elaouami8218@gmail.com (A.E.A.); md.yadri@gmail.com (M.E.-Y.); 2 Instituto de Alta Investigación, CEDENNA, Universidad de Tarapacá, Casilla 7 D, Arica 1000000, Chile; suazo.mj@gmail.com (M.J.S.); dlarozen@uta.cl (D.L.); 3 Renewable Energy and Advanced Materials Laboratory, International University of Rabat, Rabat 10100, Morocco; kawtar.feddi@uir.ac.ma; 4 Laboratoire de Chimie et Physique-Approche Multi-échelles des Milieux Complexes, Université de Lorraine, LCP-A2MC, F-57000 Metz, France; francis.dujardin@univ-lorraine.fr; 5 Centro Universitario de los Valles (CUValles), Universidad de Guadalajara, Carretera Guadalajara-Ameca Km. 45.5, Ameca, C.P. 46600 Jalisco, Mexico; maykel.courel@academicos.udg.mx

**Keywords:** intermediate band solar cells, quantum dots, power conversion efficiency

## Abstract

Solar cells that are based on the implementation of quantum dots in the intrinsic region, so-called intermediate band solar cells (IBSCs), are among the most widely used concepts nowadays for achieving high solar conversion efficiency. The principal characteristics of such solar cells relate to their ability to absorb low energy photons to excite electrons through the intermediate band, allowing for conversion efficiency exceeding the limit of Shockley–Queisser. IBSCs are generating considerable interest in terms of performance and environmental friendliness. However, there is still a need for optimizing many parameters that are related to the solar cells, such as the size of quantum dots, their shape, the inter-dot distance, and choosing the right material. To date, most studies have only focused on studying IBSC composed of cubic shape of quantum dots. The main objective of this study is to extend the current knowledge of IBSC. Thus, we analyze the effect of the shape of the quantum dot on the electronic and photonic characteristics of indium nitride and indium gallium nitride multiple quantum dot solar cells structure considering cubic, spherical, and cylindrical quantum dot shapes. The ground state of electrons and holes energy levels in quantum dot are theoretically determined by considering the Schrödinger equation within the effective mass approximation. Thus, the inter and intra band transitions are determined for different dot sizes and different inter dot spacing. Consequently, current–voltage (J-V) characteristic and efficiencies of these devices are evaluated and compared for different shapes. Our calculations show that, under fully concentrated light, for the same volume of different quantum dots (QD) shapes and a well determined In-concentration, the maximum of the photovoltaic conversion efficiencies reaches 63.04%, 62.88%, and 62.43% for cubic, cylindrical, and spherical quantum dot shapes, respectively.

## 1. Introduction

The photovoltaic device is the system that transforms solar irradiation directly into electricity, which can be after that stored into batteries for useful purposes. Numerous generations of photovoltaic devices, which are differenced by their efficiency, are commercially accessible in the market. The first-generation of the solar cells was made-up of crystalline silicon, which was inexpensive, more efficient at low temperatures, require less area, and it has been able to attain an efficiency of up to 26% [1,2,3]. In general, the main problem of the 1st generation of solar cells is the un-direct band-gap transitions implying low absorption coefficient and, therefore, high thicknesses in solar cells. The second generation, considered as thin-films cells, was cheaper than the first generation for the fact that they require fewer silicon materials; but, they shared a smaller part of the commercial market because of their lower efficiency [4,5]. The third generation was an enhancement of the previous generations in terms of performance and environmental friendliness. The central attraction of these cells is their low-cost and high-efficiency, using the unique flexibility of nanostructures to optimize absorption, carrier generation, and separation [6,7,8,9,10]. The reason behind this efficiency difference is that in the conventional photovoltaic systems, such as silicon-based p-n junctions, only photons having energy more than the energy gap are permitted to be absorbed. However, many solar spectrum regions remained unused; and, the unabsorbed photons cause an increase in device temperature, which leads to a decrease in power efficiency. Furthermore, various approaches have been suggested to improve the electron-hole generation rate, light absorption, and effectiveness of devices, like growing the energy levels numbers where more photons can be captured to produce more photocurrent, recycling of the high energy photons in multiple bands through radiative recombination, and reduction of the thermal process via capturing carries [11]. Besides that, the solar cell performance in multiple-junction devices has been improved when compared to the 33.5% limit that was proposed by Shockley and Queisser [12], Guter et al. [13], and Ameri et al. [14]. Such structures are likely to be used in spatial applications rather than terrestrial applications or even under concentration, which would reduce the required area of cell. This does not mean that this type of cells cannot be used in terrestrial application, of course, for this it is important to reduce the cost per watt peak of these structures in comparison to conventional cells belonging to the first generation and the second generations. This kind of structures is currently being studied by some groups [15,16,17,18,19] and, therefore, studies like this are valuable in providing conditions for the fabrication of device with optimal properties.

To improve the efficiency of photovoltaic cells, Barnham and Duggan [20] first theoretically proposed the idea of quantum wells (QWs) solar cells. Such a device structure is composed of a p-i-n terminal, where QWs are injected in the intrinsic part, leading to an improvement in the photocurrent density, and extending the optical absorption band beyond that of the conventional solar cell. Recently, many interesting studies concerning the multiquantum wells solar cells with some promising materials appear and they offer important performance in the conversion of solar radiations [21,22]. Another approach was made where the intermediate band (IB) was generated using quantum wires [23,24]. The newly developed method enhanced the photons absorption of the device towards the infrared region with an efficient one donor electron carrier collection. Hence, a more important short circuit current (J${}_{sc}$) is achieved, which results in higher efficiencies.

Being initially proposed theoretically, Luque and Martí [25] introduced the concept of intermediate band solar cell (IBSC), which has made a breakthrough in the standard single junction and multiple junction solar cell technology. These devices contain material with three bands, including valence band (VB), IB, and conduction band (CB). Detailed balance calculations of the ideal IBSC have suggested a theoretical power conversion of 63.2% in the case of full concentrated sunlight. Moreover, there are two main efficiency improvement strategies applied in such technology; the first one is achieved using high photons energy in the solar spectrum to produce multi exciton creation from single-photon [26,27]. In contrast, the second is mainly based on the invention of an IB within the host semiconductor bandgap that permits the absorption of photons with an energy lower than the bandgap to excite electrons of the VB into the IB, then, the absorption of a second sub-bandgap photon to excite electrons to CB within the IB [28,29]. Although extensive research for the efficiency enhancement of quantum dots (QD) solar cells has been investigated, there are still many parameters to be optimized for achieving that goal [30].

Recently, by using the Kronig–Penney model and taking into account the hole level, we have studied the photovoltaic conversion efficiency of solar cells based on the introduction of a lattice of InN/In${}_{x}$Ga${}_{1-x}$N QDs in the i-region of a p-i-n photodiode. Our results show that the performances of this new generation of solar cell increase considerably and can be adjusted by controlling the size, inter-dot spacing, and In-concentration [31]. We have also analyzed the role of the internal electric field induced by the polarization inside the active region of the p-i-n photodiode [32]. While the electric field increases, the energy levels of electrons and holes decrease. Consequently, the photo-generated current density J${}_{sc}$ decreases because the intermediate levels of electron and holes are shifted from their ideal positions as electric field increases, thereby decreasing the absorption of photons. Thus, the efficiency decreases with increasing the electric field.

To our knowledge, almost all the theoretical works dealing with the IBSC only consider cubic geometry in determining the characteristic of this new type of photodiode. In this work, we study the behavior of different parameters and characteristics of a multiple quantum dots solar cell (MQDSC) structure, when considering cubic, spherical, and cylindrical shapes, the inter dot space and alloy composition.

## 2. Theoretical Background and Mathematical Modeling

### 2.1. Electronic Characteristics

**Figure 1:**
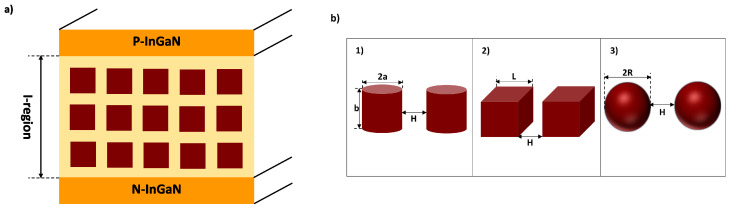
Schematic diagram of the proposed InN/In${}_{x}$Ga${}_{1-x}$N MQDSC. The red cube represents InN QDs, which are embedded into the In${}_{x}$Ga${}_{1-x}$N matrix (**a**) and different shapes of QD considered in the investigation (**b**): (1) cylindrical, (2) cubical, and (3) spherical

In the present paper, we consider a system that is composed of InN/In${}_{x}$Ga${}_{1-x}$N (well/barrier) QDs periodic array implemented in the intrinsic region of the p-i-n structure, as shown in Figure 1. Such an arrangement supports three-photon absorption in comparison with one in the conventional p-n device. The barrier semiconductor material In${}_{x}$Ga${}_{1-x}$N, which is named the host or intrinsic material, is placed between the p-n emitters and includes a periodic arrangement of semiconductor material InN QDs. The InN QDs are assumed to have three different shapes: cubic, spherical, and cylindrical structures. It is well known that the III-V semiconductors are usually used to manufacture and fabricate a new generation of solar cells among InN and GaN QDs that are particularly famous for optoeletronic devices. However, the more important thing about this class of material is that the energy bandgap of host In${}_{x}$Ga${}_{1-x}$N material can be tuned and optimized by changing the concentration *x*, which leads to an optimal energy band allowing the absorption of broader solar spectrum, leading to an enhancement in the performance of the device. The diversity in bandgap energies of both semiconductors material (InN and In${}_{x}$Ga${}_{1-x}$N) leads to an energy difference between CB and VB of these materials, which are denominated as the conduction band offset (CBO) and the valence band offset (VBO), respectively. The alternating offsets and semiconductor materials produce three-dimensional confining potential wells [33]. For the fact that a host semiconductor material entirely encloses QD nanostructure, the energy levels in the band offsets related to the QD are discrete. If the number of QDs is raised and arrayed in a periodic structure, the energy levels increase and split to produce the bands. These bands are named IBs and they are positioned inside the host semiconductor material bandgap. In such structures, regularity is necessary for the both inter dot spacing and size of QDs, because the bandwidth energies of the IBs depend on the spacing of the QDs inside the lattice and wavevector overlap [34]. In practice, most of the dots are arranged in random order or are diverse in dimension; therefore, the energy levels are irregular throughout the offsets and, hence, IBs are not produced [35]. Under these hypotheses, the discrete energy levels in the QDs are determined using the time-independent Schrödinger equation.

#### 2.1.1. Cubic Quantum Dots

We use the time-independent Schrödinger equation to obtain the discrete energy levels in the cubic quantum dots (CQD) system, which is characterized by three lengths $L_{x}=L_{y}=L_{z}=L$, and separated by three inter dot distances $H_{x}=H_{y}=H_{z}=H$ (see Figure 1(b2)), expressed as:

(1) HΨ(X,Y,Z)=EΨ(X,Y,Z).

Using the effective mass approximation, the In-concentration (*x*) dependent single-particle Hamiltonian of InN/In${}_{x}$Ga${}_{1-x}$N CQDs structure can be formulated as three independent terms:

(2) H=H(X)+H(Y)+H(Z)

where

(3) $$H\left(i\right)=\frac{-\hbar^{2}}{2m_{j}^{*}\left(x\right)}\Delta_{i}+V_{i}^{j}\left(x\right),\;\;\;\;\;\mathrm{with}\;\left\{\begin{array}{c} i=X,\;Y,\;and\;Z\\ j=e\;and\;h \end{array}\right.$$

The first term signifies the kinetic energies, and the second term describes the CBO and VBO for electron and hole, respectively, and their expressions depend on the In composition (*x*):

(4) $$V_{i}^{j}\left(x\right)=\left\{\begin{array}{cc} 0 & \mathrm{if}\;|i|\;<\;L/2\\ V_{0}^{j}\left(x\right) & \mathrm{if}\;|i|\;\geq \;L/2 \end{array}\right.$$

If we consider the well known expression of the bandgap energy of the host semiconductor material In${}_{x}$Ga${}_{1-x}$N as a function of *x* [33]:

(5) $$E_{g}\left({\mathrm{In}}_{x}{\mathrm{Ga}}_{1-x}N\right)=0.56+2.671\cdot \left(1-x\right)$$

The confinement potential in CB and VB can be expressed, as follows [36]:

(6) $$V_{0}^{j}\left(x\right)=\left\{\begin{array}{cc} 0.7\times \left[E_{g}\left({\mathrm{In}}_{x}{\mathrm{Ga}}_{1-x}N\right)-E_{g}\left(\mathrm{In}N\right)\right] & \mathrm{if}\;j=e\\ 0.3\times \left[E_{g}\left({\mathrm{In}}_{x}{\mathrm{Ga}}_{1-x}N\right)-E_{g}\left(\mathrm{In}N\right)\right] & \mathrm{if}\;j=h \end{array}\right.$$

The effective masses of the charge carrier is found by the following equations:

(7) $$m_{j}^{*}\left(x\right)=\left\{\begin{array}{cc} m_{j}^{*}\left(\mathrm{In}N\right) & \mathrm{inside}\\ x\;m_{j}^{*}\left(\mathrm{In}N\right)+\left(1-x\right)m_{j}^{*}\left(\mathrm{Ga}N\right) & \mathrm{outside} \end{array}\right.$$

Following the commutation rules, the wavevector Ψ(X,Y,Z) can be denoted as a product of three independent 1D wave functions:

(8) Ψ(X,Y,Z)=ψ(X)·ψ(Y)·ψ(Z)

and the total energy *E* is the sum of the 1D-eigenvalues, as given by:

(9) E=E(X)+E(Y)+E(Z)

The solutions of three-dimensional Schrödinger equation for the ground state energy are obtained by considering the boundary conditions given by Ben-Daniel-Duke [37,38]:

(10) $${\left[\psi_{well}\left(i\right)\right]}_{i=\pm \frac{L}{2}}={\left[\psi_{barrier}\left(i\right)\right]}_{i=\pm \frac{L}{2}}$$

(11) $$\frac{1}{m_{j}^{*}\left(\mathrm{In}N\right)}{\left[\frac{d\psi_{well}\left(i\right)}{di}\right]}_{i=\pm \frac{L}{2}}=\frac{1}{m_{j}^{*}\left({\mathrm{In}}_{x}{\mathrm{Ga}}_{1-x}N\right)}{\left[\frac{d\psi_{barrier}\left(i\right)}{di}\right]}_{i=\pm \frac{L}{2}}$$

The mixture of the two preceding equations leads to a transcendental equation [39]:

(12) $$\tan{\left(\frac{m_{j}^{*}\left(\mathrm{In}N\right)E\left(i\right)L^{2}}{2\hbar^{2}}\right)}^{1/2}={\left(\frac{m_{j}^{*}\left(\mathrm{In}N\right)}{m_{j}^{*}\left({\mathrm{In}}_{x}{\mathrm{Ga}}_{1-x}N\right)}\left(\frac{V_{0}^{j}\left(x\right)-E\left(i\right)}{E(i)}\right)\right)}^{1/2}$$

The solution of the previous equation yields the determination of the one-particle state energy E(i) in all three directions.

#### 2.1.2. Spherical Quantum Dots

Now, let us examine the case of spherical quantum dots (SQD) with radii R and separated by an inter dot distance *H* (see Figure 1(b3)). In this case, it is well known that the wave function writes as a product of radial $R_{nl}\left(r\right)$ and the spherical Harmonic $Y_{m}^{l}\left(\theta ,\phi \right)$ parts. Solving the radial equation considering the boundary conditions yields the eigenvalues of the quantized levels of both electrons and holes. Using the effective mass approximation, the radial Schrödinger part can be expressed as:

(13) $$H_{j}R\left(r\right)=E_{j}R\left(r\right),\;\;\;\;\;\mathrm{with}\;j=e\;and\;h\;$$

where the single-particle Hamiltonian that is represented in spherical coordinates can be obtained by:

(14) $$H_{j}=\frac{-\hbar^{2}}{2m_{j}^{*}\left(x\right)}\left[\frac{2}{r}\frac{\partial }{\partial r}+\frac{\partial^{2}}{\partial r^{2}}-\frac{l(l+1)}{r^{2}}\right]+V_{w}^{j}\left(r\right)$$

and the confining potential $V_{w}^{j}\left(r\right)$ is given by:

(15) $$V_{w}^{j}\left(r\right)=\left\{\begin{array}{cc} 0 & \mathrm{if}\;0\;<\;r\;<\;R\\ V_{0}^{j}\left(x\right) & \mathrm{otherwise} \end{array}\right.$$

After considering the Ben-Daniel-Duke boundary conditions at r=R, as given by:

(16) $${\left[R_{well}\left(r\right)\right]}_{r=R^{-}}={\left[R_{barrier}\left(r\right)\right]}_{r=R^{+}}$$

(17) $$\frac{1}{m_{j}^{*}\left(\mathrm{In}N\right)}{\left[\frac{dR_{well}\left(r\right)}{dr}\right]}_{r=R^{-}}=\frac{1}{m_{j}^{*}\left({\mathrm{In}}_{x}{\mathrm{Ga}}_{1-x}N\right)}{\left[\frac{dR_{barrier}\left(r\right)}{dr}\right]}_{r=R^{+}}$$

For the first state (l=0), the wave function is reduced to the spherical Bessel function of zero order [40]. The transcendental equation for the fundamental state can be formulated as:

(18) $$\begin{array}{cc} \cot{\left(\frac{2\;m_{j}^{*}\left(\mathrm{In}N\right)\;E_{n,0}\;R^{2}}{\hbar^{2}}\right)}^{1/2}= & {\left(\frac{\hbar^{2}}{2\;m_{j}^{*}\left(\mathrm{In}N\right)\;E_{n,0}\;R^{2}}\right)}^{1/2}\left(1-\frac{m_{j}^{*}\left(\mathrm{In}N\right)}{m_{j}^{*}\left({\mathrm{In}}_{x}{\mathrm{Ga}}_{1-x}N\right)}\right)\\ & -{\left(\frac{m_{j}^{*}\left(\mathrm{In}N\right)\left(V_{0}^{j}\left(x\right)-E_{n,0}\right)}{m_{j}^{*}\left({\mathrm{In}}_{x}{\mathrm{Ga}}_{1-x}N\right)\;E_{n,0}}\right)}^{1/2} \end{array}$$

Subsequently, the bound state energy eigenvalues $E_{n,0}$ are determined, where *n* is the number of radial nodes and the ground state energy is $E_{0,0}$.

#### 2.1.3. Cylindrical Quantum Dots

In this part, we consider a non-correlated electron and hole in a cylindrical quantum dot of radius *a* and height *b*, being separated by radial and z-axis inter dot distance *H*, and embedded in other semiconductor with finite potential barrier (see Figure 1(b1)). In the case of parabolic non-degenerated bands, the Schödinger equation of each particle in the effective mass approximation can be written as:

(19) $$\left(\frac{-\hbar^{2}}{2m_{j}^{*}\left(x\right)}\nabla_{j}^{2}+V_{w}^{j}\right)\Psi_{j}\left(\rho_{j},\varphi_{j},z_{j}\right)=E_{j}\Psi_{j}\left(\rho_{j},\varphi_{j},z_{j}\right)$$

This problem will be treated by following our previous approach, in which we consider that the confinement potential can be considered to be sum of two independent terms $V_{w}^{j}\left(\rho_{j},z_{j}\right)=V_{w}^{j}\left(\rho_{j}\right)+V_{w}^{j}\left(z_{j}\right)$ [41]. In these conditions, the independence of the in-plan and z-axial motions can be separated. For the in-plan motion, the confining potential is written as:

(20) $$V_{w}^{j}\left(\rho_{j}\right)=\left\{\begin{array}{cc} 0 & \mathrm{if}\;\;\rho_{j}\;\leq \;a\\ V_{0}^{j}\left(x\right) & \mathrm{if}\;\;\rho_{j}\;\geq \;a \end{array}\right.$$

and the effective mass Schrödinger equation is given by:

(21) $$\left\{\begin{array}{cc} \frac{-\hbar^{2}}{2m_{j}^{*}\left(\mathrm{In}N\right)}\nabla_{j}^{2}f_{j}\left(\rho_{j}\right)=E_{\rho j}f_{j}\left(\rho_{j}\right) & \mathrm{if}\;\;\rho_{j}\;<\;a\\ \frac{-\hbar^{2}}{2m_{j}^{*}\left({\mathrm{In}}_{x}{\mathrm{Ga}}_{1-x}N\right)}\nabla_{j}^{2}f_{j}\left(\rho_{j}\right)+V_{0}^{j}\left(x\right)f_{j}\left(\rho_{j}\right)=E_{\rho j}f_{j}\left(\rho_{j}\right) & \mathrm{if}\;\;\rho_{j}\;>\;a \end{array}\right.$$

This equation can be solved analytically, and the corresponding ground state wave functions are found to be like:

(22) $$f_{j}\left(\rho_{j}\right)=\left\{\begin{array}{cc} J_{0}\left(\alpha_{j}\rho_{j}\right) & \mathrm{if}\;\;\rho_{j}\;<\;a\\ A_{j}K_{0}\left(\beta_{j}\rho_{j}\right) & \mathrm{if}\;\;\rho_{j}\;>\;a \end{array}\right.$$

where $J_{0}$ is the Bessel function of the first kind, $K_{0}$ is the modified Bessel function of the second kind [42], $\alpha_{j}={\left(\frac{2m_{j}^{*}\left(\mathrm{In}N\right)}{\hbar^{2}}E_{\rho j}\right)}^{1/2}$, $\beta_{j}={\left(\frac{2m_{j}^{*}\left({\mathrm{In}}_{x}{\mathrm{Ga}}_{1-x}N\right)}{\hbar^{2}}\left(V_{0j}-E_{\rho j}\right)\right)}^{1/2}$, and $A_{j}$ is a constant that is obtained by considering the boundary conditions at $\rho_{j}=a$:

(23) $$A_{j}=\frac{J_{0}\left(\alpha_{j}a\right)}{K_{0}\left(\beta_{j}a\right)}$$

The ground state energy $E_{\rho j}$ in 2D motion taking the boundary conditions into consideration is a solution of the next transcendental equation:

(24) $$m_{j}^{*}\left({\mathrm{In}}_{x}{\mathrm{Ga}}_{1-x}N\right)\alpha_{j}J_{1}\left(\alpha_{j}a\right)K_{0}\left(\beta_{j}a\right)-m_{j}^{*}\left(\mathrm{In}N\right)\beta_{j}J_{0}\left(\alpha_{j}a\right)K_{1}\left(\beta_{j}a\right)=0$$

For the *z*-axis motion, the confinement is approximated by a square well that is given by the following expression:

(25) $$V_{w}^{j}\left(z_{j}\right)=\left\{\begin{array}{cc} 0 & \mathrm{if}\;|z_{j}|\;\leq \;b/2\\ V_{0}^{j}\left(x\right) & \mathrm{if}\;|z_{j}|\;>\;b/2 \end{array}\right.$$

In this case, the analytic resolution of the Schrödinger equation allows for determining the z-component of the wave function [43]:

(26) $$g_{j}\left(z_{j}\right)=\left\{\begin{array}{cc} \cos(\lambda_{j}z_{j}) & \mathrm{if}\;|z_{j}|\;<\;b/2\\ B_{j}\exp\left(\gamma_{j}z_{j}\right) & \mathrm{if}\;\;|z_{j}|\;>\;b/2 \end{array}\right.$$

with: $\lambda_{j}={\left(\frac{2m_{j}^{*}\left(\mathrm{In}N\right)}{\hbar^{2}}E_{jz}\right)}^{1/2}$, $\gamma_{j}={\left(\frac{2m_{j}^{*}\left({\mathrm{In}}_{x}{\mathrm{Ga}}_{1-x}N\right)}{\hbar^{2}}\left(V_{0j}-E_{jz}\right)\right)}^{1/2}$, and $B_{j}$ is a constant that is determined by the boundary conditions at $z_{j}=b/2$:

(27) $$B_{j}=\frac{\cos\left(\lambda_{j}\frac{b}{2}\right)}{\exp\left(-\gamma_{j}\frac{b}{2}\right)}$$

Taking the boundary conditions at $z_{j}=b/2$ into account, the axial ground state energy $E_{jz}$ can be determined by solving the following equation:

(28) $$m_{j}^{*}\left({\mathrm{In}}_{x}{\mathrm{Ga}}_{1-x}N\right)\lambda_{j}\tan\left(\lambda_{j}\frac{b}{2}\right)-m_{j}^{*}\left(\mathrm{In}N\right)\gamma_{j}=0$$

The total ground state energy for a single particle can be expressed by:

(29) $$E_{j}=E_{j\rho }+E_{jz}$$

### 2.2. Photonic Characteristics

**Figure 2:**
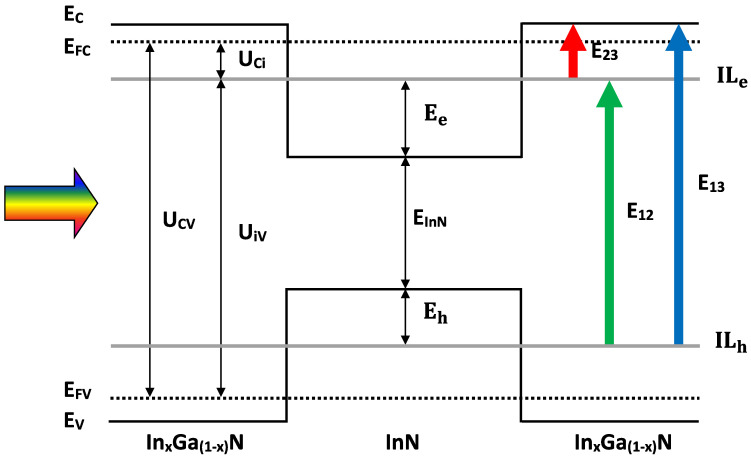
Schematic energy band diagram of the proposed InN/In${}_{x}$Ga${}_{1-x}$N MQDSC, where E${}_{e}$ and E${}_{h}$ are the energies of electron and hole, respectively; corresponding to the intermediate levels IL${}_{e}$ and IL${}_{h}$. E${}_{C}$ and E${}_{V}$ are, respectively, the energies of CB and VB.

After determining the electronic properties of the MQDSC device, it is now time to study the system from a solar cell point of view. Thus, the current density, output voltage, as well as the photovoltaic performance of the device are being determined. It is assumed that only radiative band-to-band transitions are taken into consideration; hence, photon emission and absorption are being described by the process of recombination and generation, respectively. From Figure 2, which represents the band structure of our system, we can remark the existence of three allowed energy transitions: the first transition between VB and IL${}_{e}$ (E${}_{12}$), the second between IL${}_{e}$ and CB (E${}_{23}$), alongside the third (conventional) transition that occurs between VB and CB (E${}_{13}$). The conventional energy gap transition E${}_{13}$ can be written as a sum of the independently sub-gaps energy transitions E${}_{12}$ and E${}_{23}$: E${}_{13}=\;$E${}_{12}+\;$E${}_{23}$.

We note that the flux of photons emitted or absorbed by a material is provided by Roosbroeck–Shockley relation:

(30) $$N\left(E_{l},E_{u},T,U\right)=\frac{2\pi n_{s}}{h^{3}c^{2}}\int_{E_{l}}^{E_{u}}\frac{E^{2}}{e^{\frac{E-U}{kT}}-1}dE$$

where $E_{l}$ and $E_{u}$ are the lower and upper limit energy, respectively, $n_{s}=2.1646\times 10^{-5}$ is the geometric factor, *T* is the temperature, *h* is Plank constant, *c* is the speed of light, *k* is Boltzmann constant, and *U* is the chemical potential. For the sake of simplicity, we assume that $E_{23}\leq E_{12}\leq E_{13}$. Consequently, photons having energy between E${}_{23}$ and E${}_{12}$ are being absorbed; thus, an electron is being excited from IL${}_{e}$ to CB. However, and owing to thermalization process, energy excess that is higher than E${}_{23}$ and fewer than E${}_{12}$ will be wasted, and the electron will be relaxed to the CB edges till next radiative transition occurs. Still, the value of this thermalization process is least in nanostructures when compared to bulk cases. Therefore, the same impact is produced for photons that are absorbed with energies equal to E${}_{23}$ or between E${}_{23}$ and E${}_{12}$. For the second range, the absorbed photons with energy between E${}_{12}$ and E${}_{13}$ are producing the transfer of electrons from VB to IL${}_{e}$, and leaving holes in VB. Moreover, the same behavior for excess energy is observed as the former case. Finally, and concerning the last range, absorbed photons with energy higher than E${}_{13}$ are producing the transfer of electrons from VB to CB, and leaving holes in VB.

The net photon flux is equivalent to the number of charge carrier flux received at the contact. If the charge carrier flux is multiplied by the electric charge of an electron, *q*, the current density, *J*, of the MQDSC for one IL${}_{e}$ is:

(31) $$\begin{array}{cc} J/q=[s_{c}n_{s}N\left(E_{13},\infty ,T_{s},0\right) & +\left(1-s_{c}n_{s}\right)N\left(E_{13},\infty ,T_{a},0\right)-N\left(E_{13},\infty ,T_{a},qV\right)]+[s_{c}n_{s}N(E_{23}\\ \; & ,E_{12},T_{s},0)+\left(1-s_{c}n_{s}\right)N\left(E_{23},E_{12},T_{a},0\right)-N\left(E_{23},E_{12},T_{a},U_{CI}\right)] \end{array}$$

where $s_{c}$ is a concentration factor, $T_{s}$ is temperature of sun, 6000K, $T_{a}$ is the ambient temperature of solar cell, 300K, qV is quasi-Fermi energy, and $U_{CI}$ is chemical potential between the conduction and intermediate levels. The terms in the first bracket describe the current density produced while the electrons transport from the valence band to the conduction band as typically for conventional solar cells. However, the terms in the other bracket design the current density created when the electron transfer from the intermediate band to the conduction band. In both of the bracketed terms, the MQDSCs receives radiation from the sun at the temperature $T_{s}$ and $T_{a}$, respectively, while it releases radiation at the temperature $T_{a}$ and a corresponding chemical potential. The current density of the MQDSCs is formulated according to the proper operation of the MQDSCs, which necessitates that there is no current extracted from the intermediate level; therefore, the current entering the intermediate level must be equal to the current leaving the intermediate level. Consequently, the second term in Equation (31) can be rewritten as:

The output voltage can be expressed as the difference of the chemical potentials between CB and VB, and it is provided by:

(32) $$qV=U_{CV}=U_{CI}+U_{IV}$$

In the present work, the light intensity on MQDSCs is determined by the number of suns, where one sun (or concentration factor $s_{c}=1$) denotes the standard radiation at the surface of the Earth’s atmosphere. Hence, at the surface of Earth’s atmosphere, the power density falling on a MQDSCs is $P_{in}=s_{c}\;\sigma_{s}\;T_{s}^{4}=1587.2$ W/m${}^{2}$, where $\sigma_{s}=5.67\times 10^{-8}$ W/m${}^{2}$K${}^{4}$ is Stefan’s constant. Subsequently, the full concentration would be achieved when $s_{c}=\frac{1}{n_{s}}$ = 46,296. The photovoltaic efficiency η of the MQDSC is a function of $P_{in}$; thus, it changes with the level concentration of $s_{c}$. We focus our investigation on the MQDSCs efficiencies with full concentrated light $s_{c}\times n_{s}=1$ and further compared with un-concentrated light $s_{c}=1$. Moreover, the fill factor (FF) is determined from the maximum area of the J-V characteristics under illumination, open-circuit voltage, and the short circuit current as:

(33) $$FF=\frac{V_{m}\times J_{m}}{V_{oc}\times J_{sc}}$$

where $V_{m}$ and $J_{m}$ are the operating points that will maximize the power output, $V_{oc}$ is the open circuit voltage, and $J_{sc}$ is the short circuit current density of the MQDSCs.

The photovoltaic efficiency relation of the MQDSCs is expressed as:

(34) $$\eta =\frac{V_{oc}\times J_{sc}\times FF}{P_{in}}=\frac{J_{m}\times V_{m}}{s_{c}n_{s}\sigma_{s}T_{s}^{4}}=\frac{J_{m}\times V_{m}}{s_{c}\times 1587.2}$$

## 3. Results

**Figure 3:**
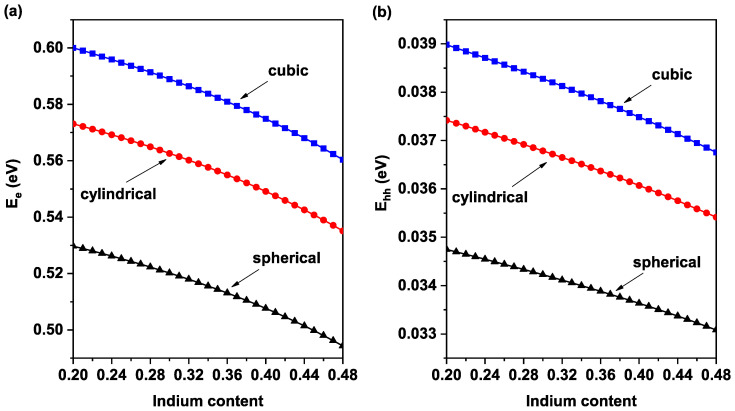
Ground state energy versus In-concentration for different QD shape: (**a**) electron and (**b**) heavy hole. Table 1 provides the dimensions of QDs.

Firstly, we start our results by discussing the confinement behavior of the different QD shape on the fundamental levels of electron E${}_{e}$ (Figure 3a) and heavy hole E${}_{hh}$ (Figure 3b). We have adopted choosing the following parameters; first, because they give the maximum efficiency, and second, for comparing different shapes, we should fix their volume. Therefore, for cubical QD, the size of the dot is fixed at L=4.5 nm. For spherical QD, the radii *R* is fixed at 2.79 nm. Lastly, for cylindrical QD, the radii *a* and the height *b* are fixed at 2.43 nm and 4.87 nm, respectively. It is worth noting that in all calculations, we have chosen an inter dot distance H=8 nm, which guarantees the non-overlapping between wave functions of QDs; thus, only discrete levels within the bandgap are created. Increasing indium concentration reduces almost linearly both fundamental levels of electron and heavy hole, regardless of QD shape, as shown in Figure 3. This behavior can be explained by the fact that In content increasing leads to a decrease in the potential barrier of both heavy hole and electron (V${}_{0}^{e}$ and V${}_{0}^{h}$). Additionally, it is observed that for all ranges of In-concentration, both electron and heavy hole are more confined in the cubic QD form, followed by the case of cylindrical QD, and lastly, spherical QD. However, even if all of the QD shape has the same volume, the confinement behavior is different, leading to the conclusion that the shape of QD affects the electronic properties of nanomaterials, something that could help in the manufacturing of optoelectronic devices with a range of features.

**Figure 4:**
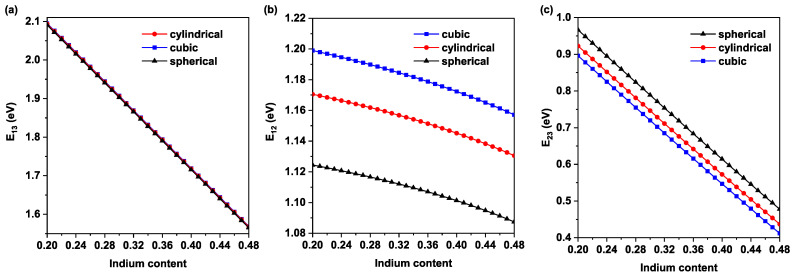
Variations of the energy transitions versus In-concentration for various QD shape: (**a**) sub-band energy transition E${}_{12}$, (**b**) host band gap E${}_{13}$, and (**c**) sub-band energy transition E${}_{23}$.

In order to complete our analysis, in Figure 4a we plot the host band gap E${}_{13}$ (VB→CB), which describes the main transition between VB and CB, as in the case of conventional solar cell devices. It is shown that this transition is slightly affected by the shape of QD, because the different form of QD leads to various close values of E${}_{hh}$. However, In-concentration leads to a decrease in the host band gap, following Equation (5). It is worthy to point out that the host band gap E${}_{13}$ is principally affected by the type of barrier material (In${}_{x}$Ga${}_{1-x}$N in our case). Thus, it is crucial to choose the appropriate material to enhance the performance of the devices. Figure 4b presents the inter-band ground state transition E${}_{12}$ between VB and IL${}_{e}$ (VB→IL${}_{e}$) versus In-concentration for different QD shape. This figure shows that the values of the E${}_{12}$ transition slightly decrease as an In content increase. Primarily, the apparent reduction of the inter-band transition energy is predominately produced by the decrease of the energy level of the electron E${}_{e}$ and heavy hole E${}_{hh}$ as In-concentration increases. Moreover, different shape leads to the distinctive behavior of the inter-band transition, which is due to the diverse response of ground state energy in various QD, as pointed out in Figure 3. Consequently, without changing the size of QD, photovoltaic devices can be modulated by varying the form of QD. The third transition E${}_{23}$ (IL${}_{e}\to$CB) versus In content and for various QD form is presented in Figure 4c. This transition is produced between IL${}_{e}$ and CB, leading to the absorption of photons of energy between E${}_{23}$ and E${}_{12}$. It is observed that different behavior from the previous figures (cubic QD, followed by cylindrical QD, and then spherical QD) is produced. Because E${}_{13}=\;$E${}_{12}+\;$E${}_{23}$, the more confinement case (cubic QD) leads to less value of the energy transition E${}_{23}$. Therefore, multiple photons with different energies can be absorbed as the form of QD is modified. However, it is shown that, as the In content increase, E${}_{23}$ decreases similarly to the behavior observed in the previous transitions.

**Figure 5:**
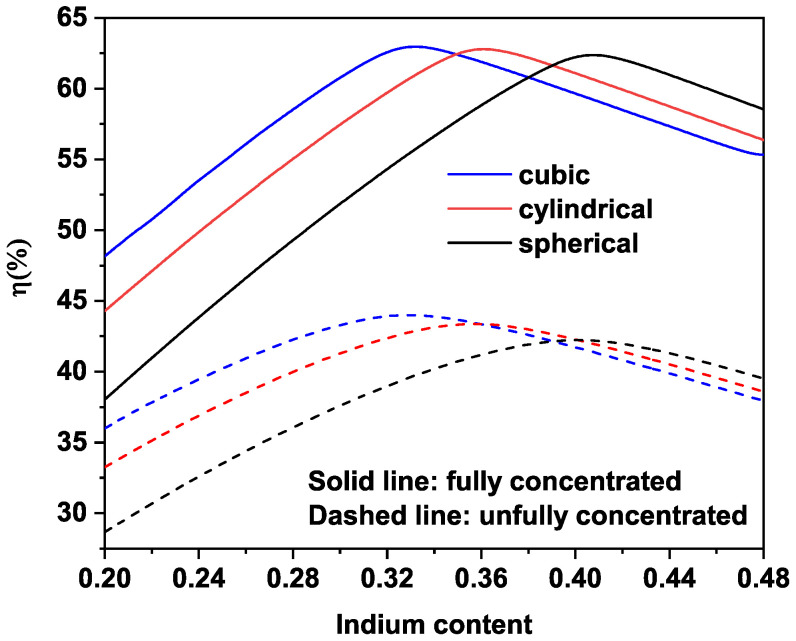
Photovoltaic conversion efficiency versus In content for various QD form: (solid lines) fully concentration $s_{c}\times n_{s}=1$, and (dashed lines) un-concentration cases $s_{c}=1$.

Figure 5 depicts the photovoltaic conversion efficiency versus In-concentration and for various QD shapes. As can be observed, η increases until a maximum value and then decreases for all the considered shape. The same behavior has been observed by many authors [25,31,44,45], and it can be explained by the fact that the increase in In-concentration affects the energy value of the IL${}_{e}$, thus influencing the transitions E${}_{12}$ and E${}_{23}$. In particular, for cylindrical MQDSC, the maximum efficiency η=62.88% is achieved for In-concentration of x=0.36. For cubic cases, the maximum efficiency of η=63.04% at a full concentrated light is reached for an In-concentration of x=0.33. Finally, for spherical QD, the maximum efficiency of η=62.43% is obtained for In content of x=0.41. Nevertheless, it is worth mentioning that the maximum efficiency is reached for the case of cubic based MQDSC for the fact that the combination of transitions (E${}_{23}=0.69$ eV and E${}_{12}=1.21$ eV) is almost the same as in the ideal case of IBSC (E${}_{23}=0.7$ eV and E${}_{12}=1.23$ eV), where a maximum efficiency of 63.2% is obtained [46], as compared to that of the cylindrical case (E${}_{23}=0.64$ eV and E${}_{12}=1.15$ eV) and spherical case (E${}_{23}=0.59$ eV and E${}_{12}=1.09$ eV). It should be noted that, under fully concentrated light, the maxima of efficiency are of the same order, but occur for different In content, while, under un-concentrated light, the maximum efficiency significantly depends on the QD shape and on the In content. Furthermore, Table 1 represents the parameters that characterize photovoltaic device performance. The primary parameters are the short circuit current density (J${}_{sc}$), the open-circuit voltage (V${}_{oc}$), and the fill factor (FF). Although, the fill factor is also a function of V${}_{oc}$ and J${}_{sc}$. Consequently, these last two parameters are the critical factors for defining the cell’s efficiency. Additionally, the numerical results shown in Table 1, which are estimated at room temperature (300 K), can be a guide for experimental fabrication of this type of solar cells.

**Figure 6:**
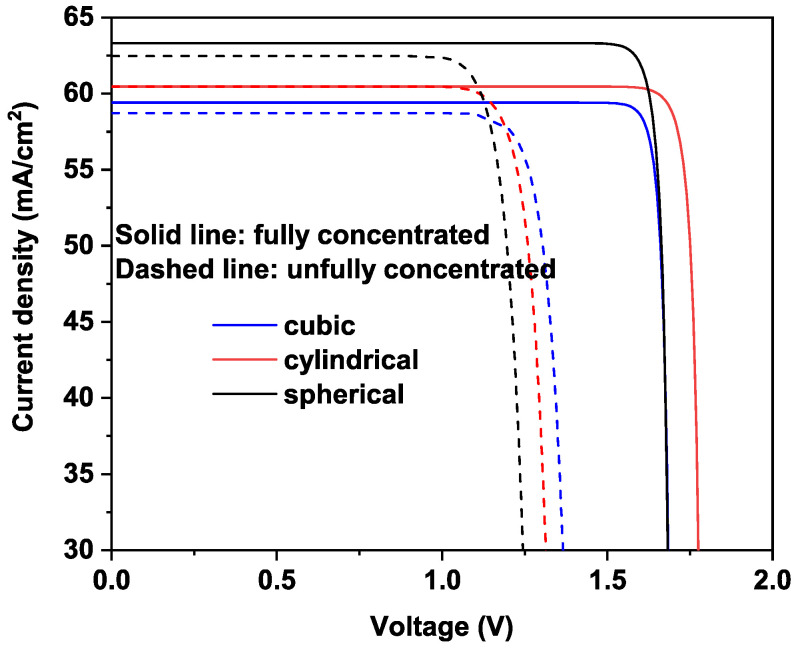
Current density versus voltage between CB and VB of the MQDSC operated at the full concentration (solid lines) and the un-concentration (dashed lines) cases. The In content is fixed at the value that maximizes efficiency.

Now let us examine the J-V characteristics of the proposed MQDSCs model. A comparison between J-V features for the full- and un-concentrated light cases, and different QD shapes are represented, as mentioned in Figure 6. It is worthy to note that the short current density is linked directly to the regime of confinement in QD; meanwhile, the open-circuit voltage is limited to E${}_{g}\left({\mathrm{In}}_{x}{\mathrm{Ga}}_{1-x}N\right)$/*q*, where *q* is the electron charge. From Figure 6, one can notice that MQDSC that is based on spherical QD denotes higher J${}_{sc}$ and small value of V${}_{oc}$ voltage as compared to other cases. However, and from a solar cell point of view, this behavior happens, because, in spherical QD cases, more photons are absorbed and exciting more electrons. Moreover, an enhanced photogenerated current is achieved; however, the small value of the output voltage prevents the enhancement of power efficiency. Besides, one can notice that, for full concentration cases, the values of open-circuit voltage V${}_{oc}$ are higher than the ones of the un-concentrated case.

**Figure 7:**
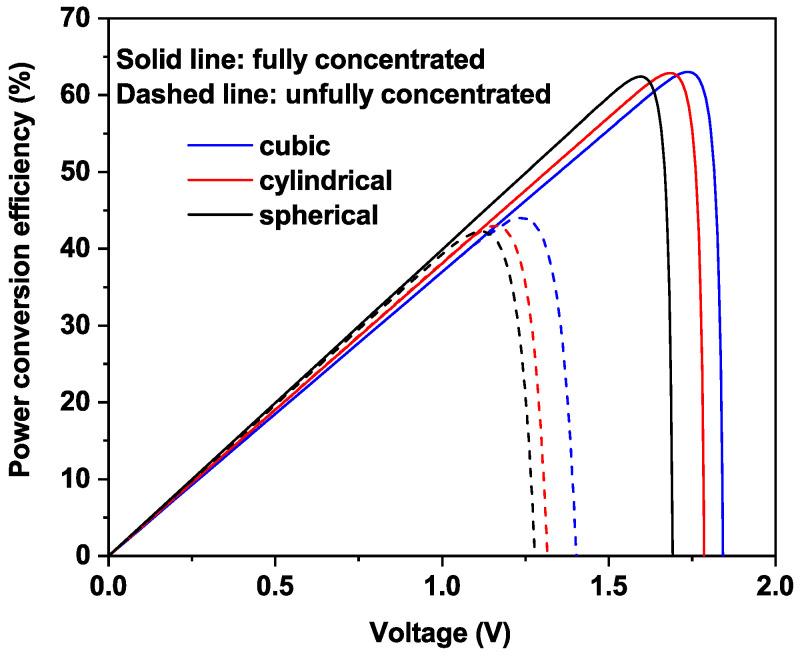
The power efficiency of the MQDSC at the full concentration (solid lines) and the un-concentration (dashed lines) cases. Note that In content is fixed at the value that gives maximum efficiency.

In Figure 7, we illustrate the photovoltaic conversion efficiency versus the voltage between CB and VB for fixed In content that achieves maximum efficiency. One can see that the obtained results, especially the behavior of the efficiency, are in good agreement with the previous studies where the maximum efficiency reaches 63.2% [47,48]. However, our new contribution concerning the comparison of different QD shape has lead to an understanding of the behavior of device power efficiency. Additionally, in the case of full concentrated light, higher values of both the current density and open circuit voltage are theoretically achieved, leading to an enhancement in photovoltaic conversion efficiency. Higher photovoltaic conversion efficiencies are obtained in the case of cubical MQDSC, followed by the case of cylindrical, and spherical MQDSCs, as shown in previous figures.

## 4. Conclusions

In this paper, we have presented a theoretical study concerning InN/InGaN multiple quantum dot solar cells that are based on parameters reported experimentally and theoretically for MQDSC model, such as the inter-dot spacing *H*, the In-concentration *x*, as well as the size of the InN-QDs by using an analytical method, within the framework of the effective-mass approximation. The impact of quantum dots shape, alongside the light concentration effect, has been investigated. This study indicates that, by changing the shape of quantum dots, the performance of solar cells is slightly modified. Moreover, it has been demonstrated that the quantum dots size, indium concentration, and light concentration play a key role in searching the maximum efficiency of multiple quantum dot solar cells. Our study provides the framework for a new way to undertake the manufacturing of such solar cell architecture. These findings add to a growing body of literature on the third generation of solar cells. Further studies, which take strain and different materials effects into account, will need to be performed.

## Figures and Tables

**Table 1:** Characteristics of the proposed MQDSC device for different shapes of QD at full-concentrated and un-concentrated light cases. The volume is fixed in all cases, and the inter-dot distance is *H* = 8nm.

	QD Shape	QD Dimensions	$x_{\max}$	V${}_{\max}$	J${}_{\max}\times n_{s}$	V${}_{\mathrm{oc}}$	J${}_{\mathrm{sc}}\times n_{s}$	FF	$\eta_{\max}$
		(nm)		(V)	(mA/cm${}^{2}$)	(V)	(mA/cm${}^{2}$)	(%)	(%)
Fully concentration	Cylindrical	a=2.43	0.36	1.68	59.23	1.78	60.45	92.47	62.88
		b=4.87							
	Cubical	L=4.50	0.33	1.74	57.48	1.84	58.72	92.56	63.04
	Spherical	R=2.79	0.41	1.59	62.06	1.69	63.31	92.22	62.43
Un-concentration	Cylindrical	a=2.43	0.36	1.18	58.12	1.34	60.45	84.66	43.38
		b=4.87							
	Cubical	L=4.50	0.33	1.23	56.58	1.40	58.72	84.65	44.02
	Spherical	R=2.79	0.40	1.11	60.61	1.27	62.46	84.81	42.24

## Data Availability

The data presented in this study are available on reasonable request to the corresponding authors.
